# Sensorineural Hearing Loss Associated with Occupational Noise Exposure: Effects of Age-Corrections

**DOI:** 10.3390/ijerph60320889

**Published:** 2009-02-26

**Authors:** Sridhar Krishnamurti

**Affiliations:** Associate Professor/Department of Communication Disorders, 1199 Haley Center, Auburn University, Auburn, AL 36849, U.S.A.; E-Mail: krishsr@auburn.edu; Tel.: +1-334-826-5258; Fax: +1-334-844-4585

**Keywords:** Occupational noise exposure, hearing loss, aging

## Abstract

Noise-induced permanent threshold shifts (NIPTS) were computed from retrospective audiometric analyses by subtracting aging effects on hearing sensitivity in sixty-eight patients with bilateral sensorineural hearing loss who reported significant occupational noise exposure histories. There were significant effects of age on NIPTS but no significant gender- or ear- differences in terms of NIPTS. The NIPTS at 2,000 Hz was found to be significantly greater than NIPTS at frequencies 500 Hz, 1,000 Hz, 4,000 Hz, and 8,000 Hz. Defined noise notches were seen in the audiograms of 38/136 (27%) ears with SNHL. Results support models that suggest interactive effects of aging and noise on sensorineural hearing loss in ears with occupational noise exposure.

## 1. Introduction

Occupational noise-induced hearing loss (NIHL) is defined as bilateral sensorineural hearing loss that develops slowly over a period of several years as the result of exposure to continuous or intermittent loud noise in the workplace. Tinnitus and NIHL have been commonly reported in military personnel who are routinely exposed to occupational noise [1]. Estimates suggest that large numbers (approximating between 5 and 30 million) Americans are exposed to hazardous noise levels in the workplace [2]. Based on exposure levels, about one out of every four workers will develop permanent hearing loss [3]. Occupational noise-induced hearing loss can significantly influence worker communication and safety and can have a tremendous impact on the lives of workers [4]. Typically, the first sign of hearing loss from noise exposure is a notching of the audiogram at 3,000, 4,000, or 6,000 Hz, with recovery at 8,000 Hz [5]. In early stages of NIHL, the average hearing thresholds at 500, 1,000, and 2,000 Hz are better than the average at 3,000, 4,000, and 6,000, and the hearing level at 8,000 Hz is usually better than the deepest part of the notch. This notch is in contrast to age-related hearing loss, which also produces high frequency hearing loss, but in a down-sloping pattern without recovery at 8,000 Hz.

Presbyacusis or Age Related Hearing Loss (ARHL), reflects the loss of hearing sensitivity associated with advanced aging and is the third most common chronic condition reported by the elderly people [6]. The typical audiometric profile observed clinically in presbycusis is a bilateral symmetric high-frequency sensorineural hearing loss that progresses with advancing age. In a study by Cruickshanks *et al.* pure tone thresholds of 3,753 adults from four age-groups (49–59 years, 60–69 years, 70–79 years, and 80–89 years) showed that: a) the average hearing thresholds in men are typically poorer than those of women in the high frequencies, b) men exhibited a sharply sloping hearing loss in the moderately severe range in the high frequencies, and c) women exhibited a more gradual sloping hearing loss in the moderate range in the high frequencies [7].

One of the limiting factors that impacts differential diagnosis and allocation of sensorineural hearing loss in the elderly is that typically age-related hearing loss tends to be confounded by previous effects of noise exposure in those individuals employed previously in a noisy workplace environment. Sensorineural hearing loss related to noise exposure typically does not produce a loss greater than 75 decibels (dB) in high frequencies and 40 dB in lower frequencies [8]. Noise-induced hearing losses along with superimposed age-related losses may have hearing threshold levels in excess of these values.

Since age-related hearing loss and noise-induced hearing loss progress simultaneously, audiometric testing cannot be used to separate these effects. Noise-induced permanent threshold shifts (NIPTS) can be determined from retrospective audiometric analyses by subtracting aging effects on hearing sensitivity. Several workers compensation programs that follow the Occupational Safety and Health Agency (OSHA) standard [9] apply age-corrections by subtracting a decibel value based on the person’s age from the measured audiometric thresholds to document possible handicap due to noise exposure. It is not clear how NIPTS is influenced across younger- and older listeners, if such age-corrections are indeed applied to individual audiograms.

### 1.1. Purpose

There were three primary aims of the retrospective research analyses conducted in the current study. The first aim was to investigate if there are significant differences in the degree of NIPTS after correcting for age-effects in younger and older individuals with occupational noise exposure. The second aim was to investigate possible gender- and ear- related effects on NIHL. The third aim was to explore if there were differences between audiometric frequencies in terms of the degree of NIPTS and if occurrence of noise notches differed in younger and older individuals with sensorineural hearing loss.

### 1.2. Methods

For the purpose of this study, audiometric findings of sixty-eight successive patients with bilateral sensorineural hearing loss and significant noise exposure histories evaluated between June 1, 2006 and May 30, 2007 were selected for retrospective analyses. Subjects were patients of a hospital-based audiology clinic located in northeastern AL. Hearing thresholds in 68 successive patients collected over a one year period between June 1, 2006 and May 30, 2007 at frequencies 500, 1,000, 2,000, 4,000, and 8,000 Hz were first compiled from both right and left ears. All subjects were required to show significant histories of occupational noise exposure and bilateral symmetrical sensorineural hearing losses to be included in the analyses. Subjects with asymmetrical hearing losses, air-bone gaps, or other significant medical histories were excluded from analyses.

**Figure 1.**
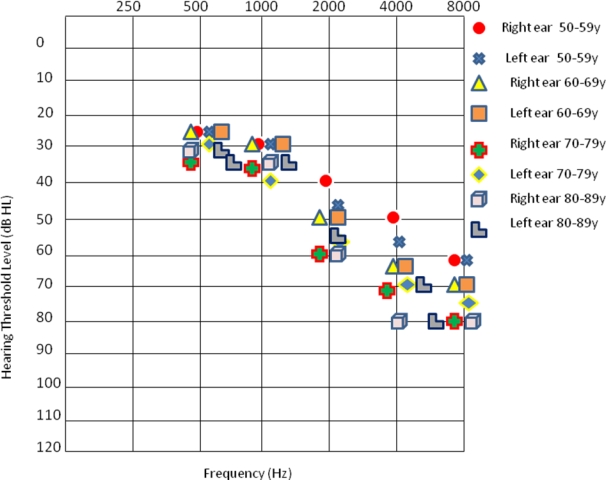
Mean audiograms of subjects from four age-groups included in this study.

All subjects were classified into four age-groups: 1) 50–59 years, 2) 60–69 years, 3) 70–79 years, and 4) 80–89 years. More details related to the subject population are included in Table 1. The mean audiometric thresholds of subjects from younger and older subjects with occupational noise exposure (without applying age-corrections) are shown in Figure 1.

The effects of occupational noise exposure were further evaluated by applying age- and gender- related corrections based on mean age-related norms (obtained from the Cruickshanks *et al.* study) [7]. Individual corrections were carried out for each subject by subtracting thresholds based on equivalent age and gender data (obtained from Table 3 of the Cruickshanks *et al.* article) from the actual audiometric thresholds of the individual subject.

Noise dose measures (typically used to characterize risk of NIHL) could not be obtained in this study which was clinical and retrospective by design. However, mean number of years of occupational noise exposure (see Table 1) showed equivalent noise exposure across age-groups. Also audiometric thresholds (without age-correction) that are shown in Figure 1 showed similar findings across age-groups. Hence younger and older listeners seemed to have similar noise exposure and findings prior to age- and gender- corrections.

Analyses of noise notches for audiograms were conducted by an expert panel of three independent judges. Previous studies have shown excellent agreement among experts on ‘notch identification’ [10]. For the purposes of analysis, a noise notch was defined based on previously published Coles *et al.* [11] criteria: a) presence of elevated thresholds in the 3–6 kHz region of the audiogram, b) hearing loss in frequencies 3–6 kHz at least 10 dB worse than the worst hearing threshold values at 500 Hz or 1 kHz, and c) hearing thresholds at 8 kHz at least 10 dB better than the worst threshold at 3, 4, or 6 kHz. Such high frequency audiometric notches with relatively better hearing at lower frequencies and recovery in the audiogram at 8 kHz have been considered typical of NIHL [12]. Due to the retrospective clinical nature of the study design, audiometric threshold data were not available for all patients at frequencies 3 kHz and 6 kHz. Hence modification of the Coles *et al.* [11] criteria were used as follows: a) presence of elevated thresholds in the 4 kHz region of the audiogram, b) hearing loss at 4 kHz at least 10 dB worse than the worst hearing threshold values at 500 Hz, 1 kHz, or 2 kHz and c) hearing thresholds at 8 kHz at least 10 dB better than the worst threshold at 4 kHz.

## 2. Results

Age- and gender- corrected NIPTS data obtained at audiometric frequencies on both ears of all subjects above were statistically analyzed to study the effects of age, gender, ear, and audiometric frequency. Multivariate analyses of variance (MANOVA) were conducted on NIPTS data obtained above to evaluate the effects of various factors (age, gender, ear, and audiometric frequency) with repeated measures on the audiometric frequency factor. Analysis of noise notches were made in each ear in the audiograms of all 68 subjects (136 ears) based on criteria defined above.

### 2.1. NIPTS across Age Groups

**Figure 2.**
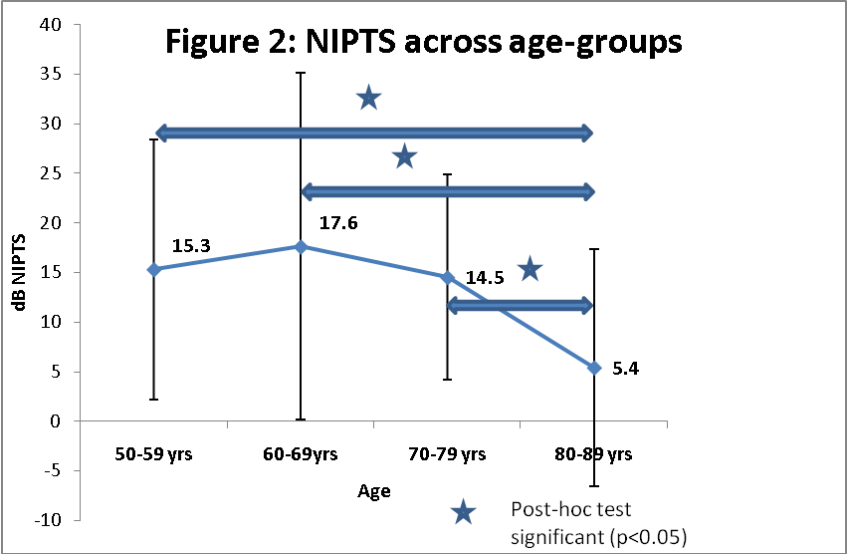
NIPTS across age-groups after age-corrections.

Age-corrected data obtained above were subjected to multivariate analyses of variance (Table 2) based on various factors (age, gender, and ear). There were significant effects of age on noise-related threshold shifts {F (3, 120) = 4.84; p<0.01)}. The mean NIPTS and standard deviations (error bars) for each of the age-groups are shown in Figure 2. Post-hoc (Newman-Keuls) analyses were carried out to look for significant differences between age-groups and results showed that three age groups (50–59 years; 60–69 years; 70–79 years) showed significantly greater amounts of NIPTS than the oldest (80–89 years) age-group (see Figure 2).

### 2.2. NIPTS across Ear and Gender

There were no significant ear-differences (right versus left) across age-groups and gender (See table 2). No significant gender differences were found in NIPTS from male and female listeners in the same age group.

### 2.3. NIPTS across Audiometric Frequencies

**Figure 3.**
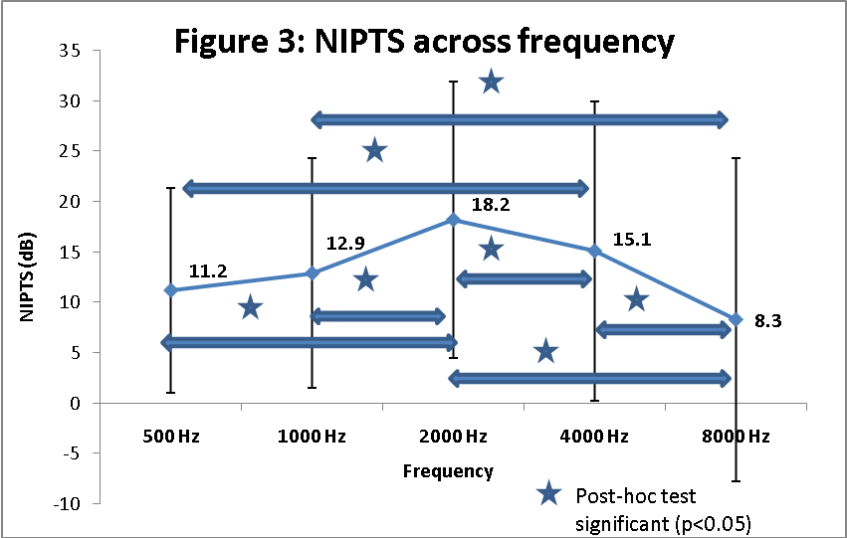
NIPTS across audiometric frequencies after age-corrections.

Greatest noise-related threshold shifts were seen at 2,000 Hz (re: 500 Hz, 1,000 Hz, 4,000 Hz, 8,000 Hz) on post-hoc analyses (see Figure 3). In the current study, noise notch determination based on review of audiograms of patients with occupational noise exposure showed excellent (>90%) agreement among experts. A total of 32/68 (47%) subjects showed noise notches in the current retrospective study. For purposes of analysis of noise notches, both ears of each of the 68 subjects were included (total of 136 ears). Defined notches were seen in the audiograms of 38/136 (27%) ears with SNHL. A statistically significantly greater number of notches (χ^2^ = 201.44; df = 1; p<0.01) were seen in the left ear (22/136 or 16%) when compared with those in the right ear (16/136 or 11%).

## 3. Discussion

### 3.1. NIPTS across Age

It is widely accepted that aging (presbyacusis) and noise (NIHL) are the most common causes of adult SNHL. According to the American Academy of Otolayngology-Head and Neck Surgery [13], aging and noise exposure are the common causes of sensorineural hearing loss and one in 10 Americans has a hearing loss that affects speech understanding ability. Statistics reported for NIHL indicate that about thirty million workers are at risk for NIHL and 10 million Americans already have NIHL [14]. However, results of recent studies indicate that age-related SNHL is still the most prevalent type and occupational NIHL accounts for less than 10% of the burden of adult hearing loss in the United States [15].

Following corrections applied for age and gender to individual subjects, greatest NIPTS values were obtained for three age groups (50–59 years; 60–69 years; 70–79 years) when age corrections were applied for hearing loss. Age corrections applied to individual audiograms significantly reduced the degree of NIPTS for the oldest age-group (80–89 years). Results of this study indicate that occupational noise exposure has significantly greater impact on the hearing sensitivity of listeners from three groups (50–59 years, 60–69 years, 70–79 years) with SNHL than on hearing sensitivity of the oldest age group (80–89 years). Based on these results, it clearly appears that there is an age-noise interaction which is not equally distributed across age-groups. The age-noise interaction found in the current study can be compared to interactions reported in previous animal and human studies.

In the Kujawa and Liberman animal study [16], mice of different ages (4–124 weeks) were exposed to noise (8–16 kHz; 100 dB SPL) for 2 hours and compared with cohorts (with- and without- noise exposure) over different post-exposure times (2–96 weeks). Younger mice (4–8 weeks old) mice showed threshold (40–50 dB) shifts on Auditory Brainstem Response and otoacoustic emissions tests while older cohorts (96 weeks old) with the same noise exposure did not show such threshold shifts. Also, mice with previous noise exposure that aged over time showed considerably larger threshold shifts than aging animals without noise exposure. Based on these findings, the authors concluded that in the animal model, NIHL varied across age and NIHL exacerbated Age-related hearing loss. The results of the current study disagree with this animal finding and showed that aging humans with previous noise exposure did show NIPTS but this effect decelerated over time (least NIPTS prevalent in the oldest age-group). The reasons for this difference are not clear but clearly the aging effects of human ears with previous noise exposure cannot be simulated by a simple animal model.

In the Framingham human cohort study [17], 406 audiograms from 203 older males were classified individually according to pure-tone thresholds in the 3- to 6-kHz region (the notch region). The authors found that subjects with notched thresholds had more threshold shift at frequencies immediately below the notch in the following 15 years than those with no notched thresholds. The authors assumed the notched thresholds were the result of noise exposure. Because the differences in threshold shifts mainly occurred in the regions adjacent to the notch region, the authors suggested that the effect of noise on pure-tone thresholds could continue long after the noise exposure has stopped. The current study supports this speculation because significant NIPTS were seen in all age groups even though the effect of NIHL decelerated over age (least NIPTS seen for oldest age-group).

### 3.2. NIPTS across Gender

In the current study, no significant gender effects were seen for NIPTS, i.e., both men and women showed equivalent NIPTS. Hence noise does not appear to impact men and women differentially, if both sexes are exposed to occupational noise levels. On the other hand, aging does influence males and females differentially. In a human study by Lee *et al.* [18], audiometric changes were compared longitudinally in male and female subjects ranging in age from 60–81 years. The rate of change for hearing thresholds increased significantly at an average of 1 dB per year above the age of 60 years. After adjusting for age, females showed a significantly faster rate of change in high frequencies (6–12 kHz) than males. The rates of threshold change with age were not significantly different in subjects with a history of noise exposure than subjects without noise exposure.

### 3.3. NIPTS across Audiometric Frequencies

Diagnosis of NIHL is based on: a) case history of previous or current occupational and recreational noise exposure and b) audiometric review. Audiometric losses are expected in the higher frequencies of 3, 4, or 6 kHz, where the ear is more susceptible to noise [8], a noise notch typically means thresholds at 3, 4, and/or 6 kHz that are substantially worse than hearing thresholds at lower frequencies (0.5 and 1 kHz) and at 8 kHz (where a recovery is said to take place). Several mechanisms have been offered to explain the extra vulnerability of these higher frequencies to the damaging effects of intense noise. These mechanisms include better transmission of the higher frequencies through the outer and middle ears to the inner ear [19,20] and specific vascular [21] or metabolic [22] damage of the basal regions of the cochlea. Bohne and Harding [23] showed that early noise damage was seen in the form of loss of outer cells in the basal (4–8 kHz) regions of the Organ of Corti in the chinchilla cochlea. With continued exposure, the damage progressed to loss of cell segments along the entire Organ of Corti and loss of myelinated auditory nerve fibers.

The mean NIPTS values obtained after age-correction in the current study (see Figure 3) at frequencies 1 kHz (13 dB), 2 kHz (18 dB) and 4 kHz (15 dB) were considerably greater than NIPTS predicted by models from previous reports by Dobie [24]. The reasons why NIPTS obtained in the current study differed from these predictions can be explained by methodological differences between the Dobie and current studies. In the Dobie study, median NIPTS values were estimated by calculating the median differences between threshold distributions for noise-exposed and control (non-exposed) groups. On the other hand, individual audiograms obtained from a clinical sample of patients in the current study were individually corrected for age and gender according to aging demographic data.

Noise notches were seen only in 38/136 (27.9%) ears with SNHL in the current study. This finding indicating that only about one-quarter of ears with noise exposure showed noise notches supports previous studies reporting limited frequency of noise notches individuals with occupational noise exposure. It is widely recognized that a noise notch is not a ‘prima facie’ evidence of NIHL [17] and can be affected by changes in hearing either at the frequencies most susceptible to noise (3–6 kHz) or at frequencies below or above these frequencies (500 Hz, 1 kHz or 8 kHz). It is well documented that aging effects can increase high frequency hearing loss in a down-sloping pattern without recovery at 8,000 Hz [6,17], thereby influencing characteristics of the noise notch. It is also possible that the noise exposure reported by some of the subjects may not have been high enough in level or long enough in duration to produce significant or deep noise notches. Noise notches were found in about one-half of the subjects in the current study and these findings are similar to the percentage of noise notches (57%) of the subjects those reported in Gates study [17].

### 3.4. Contributions of Aging and NIHL

Results of this study support assumptions from Corso’s model [25] speculating that presbyacusis and noise exposure do not contribute equally to permanent hearing loss over the lifespan. According to Corso’s variable ratio model [25], due to the variable interactions of aging and noise exposure over the lifespan, the contributions of these factors cannot simply be added or yield a fixed ratio over the life span. Instead, Corso [25] argues that at different age levels, the relative contributions of aging and noise to SNHL will generate a variable ratio. The variable ratio can then be used to create an age correction factor for use in any formula calculating percentage of hearing loss. Results of this study show that for younger listeners (<69 years of age), NIHL appears to be the dominant factor. On the other hand, aging (presbyacusis) appears to be the dominant factor for older listeners (>70 years of age). It appears that the effect of NIHL decelerates with age while the effect of aging accelerates over the extended life span.

## 4. Conclusions

Results of the current study indicate that the effects of noise exposure on hearing varied across age-groups and highlight the importance of applying age- and gender- corrections prior to determining the relative contribution of occupational noise exposure in patients with SNHL. More research needs to address the relative weighted contributions of aging and noise effects in the occupation NIHL population.

## Figures and Tables

**Table 1.** Subject characteristics.

Age group	Number of subjects	Mean number of years of occupational noise exposure	Gender
50–59 y	13	22 y	9 males 4 females
60–69 y	22	23 yrs 6 months	17 males 5 females
70–79 y	22	23 y 3 months	18 males 4 males
80–89 y	11	23 y 8 months	8 males 3 females

**Table 2.** MANOVA results showing effects of age, gender, ear, and audiometric frequency on NIPTS.

Effect	dF	MS effect	MS error	F	P
**Age**	3	2846.30	587.51	4.84	0.003*
**Gender**	1	86.38	587.51	0.15	0.71
**Ear**	1	532.00	587.51	0.91	0.34
**Frequency**	4	792.23	150.23	5.27	0.0003*
**Age X Gender**	3	382.640	587.51	0.65	0.58
**Age X Ear**	3	51.22	587.51	0.09	0.96
**Age X Frequency**	12	315.53	150.22	2.10	0.015*
**Gender X Ear**	1	5.00	587.51	0.008	0.93
**Gender X Frequency**	4	314.23	150.22	2.09	0.08
**Ear X Frequency**	4	30.89	150.22	0.20	0.93
**Age X Gender X Ear**	3	165.14	587.51	0.28	0.84
**Gender X Ear X Frequency**	4	51.22	587.51	0.09	0.96
